# Epilepsy in Adults with Supratentorial Glioblastoma: Incidence and Influence Factors and Prophylaxis in 184 Patients

**DOI:** 10.1371/journal.pone.0158206

**Published:** 2016-07-20

**Authors:** Shuli Liang, Junchen Zhang, Shaohui Zhang, Xiangping Fu

**Affiliations:** 1 Department of Neurosurgery, Chinese PLA General Hospital, Beijing, 100853, China; 2 Department of Neurosurgery, First Affiliated Hospital of PLA General Hospital, Beijing, 100048, China; 3 Department of Neurosurgery, Affiliated Hospital of Jining Medical College, Jining, 272029, China; Columbia University, UNITED STATES

## Abstract

**Aim:**

To analyze the incidence of epilepsy in adult patients with supratentorial glioblastoma, assess the factors influencing the development of epilepsy in these cases, and evaluate patients’ response to antiepileptic drugs (AEDs) in a series of 184 patients.

**Methods:**

We retrospectively analyzed the 184 adult patients diagnosed with supratentorial glioblastoma. All subjects were treated within our hospital and subsequently died between 2003 and 2013. The incidence of epilepsy was assessed before and after initial resection and reexamined every 2 months thereafter. We evaluated the efficacy of prophylactic AEDs in this patient population based on the gathered incidence data.

**Results:**

Of 184 patients, 43 (23.37%) were diagnosed with epilepsy before their initial resection. The total incidence of epilepsy (both pre- and postoperative) was 68.48%. The prevalence of active epilepsy reached over 80% in patients with epilepsy and survival of greater than 13 months postoperatively. Patients with glioblastoma in the frontal and/or temporal lobes had a higher prevalence of epilepsy. In the 43 patients with preoperative epilepsy, total resection of glioblastoma resulted in significantly lower seizure frequency. Patients who received epilepsy prophylaxis with AEDs for at least 6 months had significantly fewer seizures and higher Karnofsky scores than those receiving AEDs for less than one month or not at all.

**Conclusion:**

The incidence of epilepsy in adult patients with glioblastoma was high and responded poorly to AEDs in the short term. However, when taken for longer periods, AEDs can reduce the frequency of seizures in patients with glioblastoma.

## Introduction

Glioblastoma multiforme (GBM) is the most common primary intracranial tumor, and is characterized by aggressive growth and high patient morbidity.^1^ Extensive resection of intracranial tumors is challenging and collateral damage to normal brain tissue is a significant risk. Although research continues to provide progress, the long-term survival of patients with GBM is rarely a reality due to frequent tumor recurrence. [1–2]

Epilepsy is common complication of glioma that can significantly decrease patient functioning and quality of life. [3–4] The low incidence of epilepsy in GBM compared to low grade gliomas is frequently reported in the literature. [1, 5–8] However, epilepsy, when present, can be an important marker of tumor progression in GBM patients. [9] Reports on the cumulative incidence of epilepsy throughout the entire progression of GBM, from initial presentation to eventual death from the disease, are rare. [10] We present a retrospective analysis of patients who were diagnosed, treated, and died from GBM at our institution between January 2003 and December 2013. We analyze the incidence of epilepsy, the factors influencing its development, and its response to AEDs.

## Materials and Methods

### Patients

Records of patients treated at our institution between January 2003 and December 2013 were reviewed for eligibility. Patients who meet all of the following criteria were included in the study group, (1) pathological diagnosis of GBM (WHO Grade IV); (2) surgical treatment, chemotherapy, and epilepsy managed entirely within our unit; (3) death between January 2003 and December 2013 in order to assess patients’ entire course; (4) patients aged 18 years or older at the time of pathological diagnosis; (5) presentation of likely GBM 3–8 weeks prior to first operation; (6) patients with supratentorial GBM localizations; (7) underwent at least one resection which removed over 50% of tumor volume, followed by adjuvant radiotherapy and thermotherapy.

Patients who met the following criteria were excluded from the study: (1) postoperative intracranial hemorrhage, cerebral infarction, or intracranial infection; (2) the appearance/diagnosis of multiple lesions pre- or postoperatively; (3) incomplete or missing medical records and/or incomplete chemotherapy, radiotherapy, and/or AED use; (4) less than 2 months between 2 consecutive operative procedures; (5) and patients who experienced epileptic seizures before diagnosis of GBM. This study was approved by the Institutional Ethics Committee of the First Affiliated Hospital of Chinese People’s Liberation Army General Hospital and adhered to all international ethical standards and practices. All patients provided written informed consent prior to their death.

### Glioblastoma and antineoplastic treatments

The size of the GBM was determined by the maximum diameter measured on magnetic resonance imaging (MRI). For classification purposes, lesions were divided into categories based on their main anatomical location, including thalamus, bilateral, frontal, temporal, parietal, and occipital.

Tumor resection and adjunct chemo-radiotherapy were the treatments of choice for included patients. Outcome following tumor resection was graded based on the ratio of preoperative to postoperative volume on MRI. The resultant categories were gross total resection (100%), major resection (≥ 80%), and partial resection (50–79%). Methods of radiotherapy included I^131^ intra-tumor radiotherapy (30 mCi per time, every 1–3 months) and 3D-conformal radiotherapy (2 Gy × 25~30 times). Chemotherapies included intravenous nimustine (2~3mg/kg) and oral temozolomide (150-200mg/m^2^/d for 5 days).

### Epilepsy and prophylactic AED therapy

Video electroencephalography (EEG) was performed when patients presented with their first seizure. Epilepsy diagnosis was based on seizure semiology and interictal epileptic spikes on video EEG monitoring. All patients diagnosed with epilepsy were treated with AEDs.

Seventy-three patients without preoperative epilepsy received prophylactic AEDs following initial tumor resection. As this is a retrospective study, these patients were administered prophylaxis on a case-by-case basis based on the practice of individual physicians.

The AEDs used included valproate (20-25mg/kg), levetiracetam (20-30mg/kg), and oxcarbazepine (15-20mg/kg). Although single drug treatment was the preferred approach, multiple AEDs per patient were used as appropriate when one drug alone did not adequately control seizures.

In cases of short term AED administration, the drug was stopped over a 2 week period by decreasing the dose by half each week. In patients who received long term AED administration, a slower tapering was preferred and the drug was decreased by 1/3 each week for three weeks.

### Follow-up and analysis

Patients were seen in clinic every 2 months beginning 1 month after initial resection until death. Patients’ seizures, incidence of epilepsy, and quality of life were evaluated at each follow-up. The incidence of epilepsy at any given time was defined as a cumulative number of patients with epilepsy before this time divided by the entire sample size. The incidence of epilepsy in surviving patients at any given time was defined as the total number of surviving patients with epilepsy divided by the total number of surviving patients. Any incident case of epilepsy between two follow-up appointments was defined as new onset. For the purposes of this study, active epilepsy was defined as at least one seizure between two follow-up appointments. Karnofsky scores were calculated at the 12 month follow-up for patients who survived between 18 and 24 months following initial resection.

### Statistics

Statistical analyses were performed using SPSS (Version 18.0; SPSS Inc., Chicago, IL). Outcomes were described by percentage and the standard deviation of the mean. Quantitative data were analyzed using an F-test or t-test. Qualitative data were analyzed using Personal Chi-square tests or Kruskal-Wallis tests. The Kruskal-Wallis test and F-test were adopted for analysis of variance between groups. Results were considered significant for values of *P* < 0.05.

## Results

### Pre-resection data

This cohort included 184 adults with GBM. Of the 184 subjects, 100 were male (54.3%) and 84 were female (45.7%) (Table 1). The average age at first resection was 49.08±10.59 years (range: 20–69 years). Of the tumors, 20 (10.9%) were located mainly within the thalamus, 61 (33.2%) in the frontal lobe, 38 (20.7%) in the temporal lobe, 26 (14.1%) in the parietal lobe, 13 (7.1%) in the occipital lobe, and 26 (14.1%) were considered midline or bilateral (Table 2). The maximum GMB diameters ranged from 3.6 cm to 10.8 cm (mean: 6.65±1.76 cm).

**Table 1:** Patient Characteristics.

Classification	Patients with Preoperative Epilepsy	Patients with New Onset Postoperative Epilepsy	Seizure-Free Patients	*P* Value
No. of Patients	43	83	58	--
Male	22 (51.2%)	54 (66.1%)	23 (46.7%)	--
Female	21 (48.8%)	29 (34.9%)	35 (60.3%)	--
Age (years)	46.86±14.99	47.76±13.31	50.67±10.48	0.2752
Tumor Diameter (cm)	6.53±2.06	6.48±1.71	6.99±1.55	0.2077
Postop. Survival Time (months)	16.41±7.74	15.53±6.77	17.66±7.90	0.7237
Mean Recurrence Time After Initial Resection (months)	6.65±2.82	6.45±3.28	7.67±3.38	0.0715

**Table 2:** Resection and Recurrence by Lesion Location.

Tumor Location	Patients with Preoperative Epilepsy			Patients with New Onset Postoperative Epilepsy			Seizure-Free Patients		
	n	GTR	RT	n	GTR	RT	n	GTR	RT
Midline/Bilateral	2 (4.7%)	0 (0.0%)	6.00±1.41	9 (10.8%)	1 (2.9%)	6.56±2.83	15 (25.8%)	5 (17.2%)	8.13±2.80
Thalamus	2 (4.7%)	0 (0.0%)	6.00±5.66	9 (10.8%)	1 (2.9%)	6.33±3.12	9 (17.2%)	0 (0.0%)	6.11+2.89
Frontal lobe	12 (27.9%)	6 (30.0%)	6.00±3.02	38 (41.0%)	16 (47.1%)	6.82±3.70	11 (19.0%)	8 (2.9%)	8.00±3.38
Temporal lobe	18 (37.2%)	12 (60.0%)	7.50±2.50	15 (18.1%)	12 (35.3%)	5.67±2.87	5 (8.6%)	5 (17.2%)	7.40±4.22
Parietal lobe	9 (20.9%)	2 (10.0%)	6.11±2.98	9 (10.8%)	2 (5.9%)	6.56±2.79	8 (13.8%)	4 (13.8%)	7.25±4.40
Occipital lobe	0 (0.0%)	0 (0.0%)/	--	3 (3.6%)	2 (5.9%)	5.33±4.51	10 (17.2%)	7 (24.1%)	7.20±3.12
**Total**	**43**	**20 (46.5%)**	**6.65±2.82**	**83**	**34 (41.0%)**	**6.45±3.28**	**58**	**29 (50.0%)**	**7.67±3.38**

GTR, gross total resection (P = 0.5570); RT, recurrence time (months; P = 0.0715).

### Anti-neoplastic treatments and survival time

Each patient underwent between 1 and 3 operative resections (mean: 2.06±0.67), and received their first adjunct thermotherapy or radiotherapy within 2 weeks following surgery. Eighty-six patients (46.7%) received repeat I^131^ intra-tumor radiotherapy with a mean number of radiotherapy treatments of 3.14±0.72 (range: 2–5). Ninety eight (53.3%) patients underwent 3D-conformal radiotherapy. The chemotherapy drug nimustine was used in 99 cases (53.8%), with temozolomide in the remaining 85 cases (46.2%). Survival time ranged from 4–35 months (median: 15 months; mean 16.41±7.39 months).

### Incidence of epilepsy and seizure types

**Fig 1:**
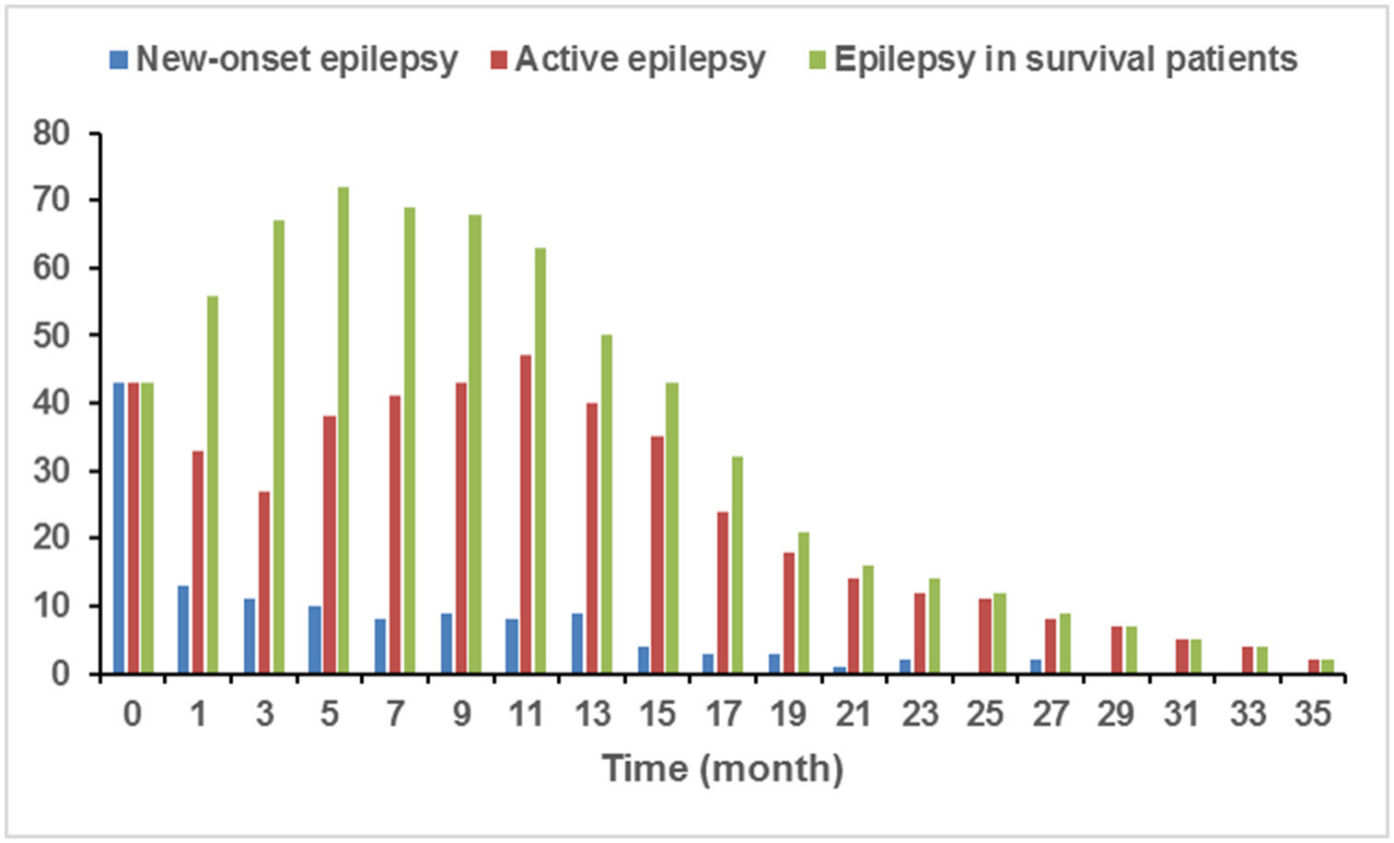
The Incidence of Epilepsy in Patients with GBM. The number of epilepsy cases in patients with GBM before initial resection (month 0), the number of patients with new onset epilepsy (red), and the number of patients with active epilepsy (yellow), and the total number of patients with epilepsy (blue) are shown at throughout disease progression following initial resection.

**Fig 2:**
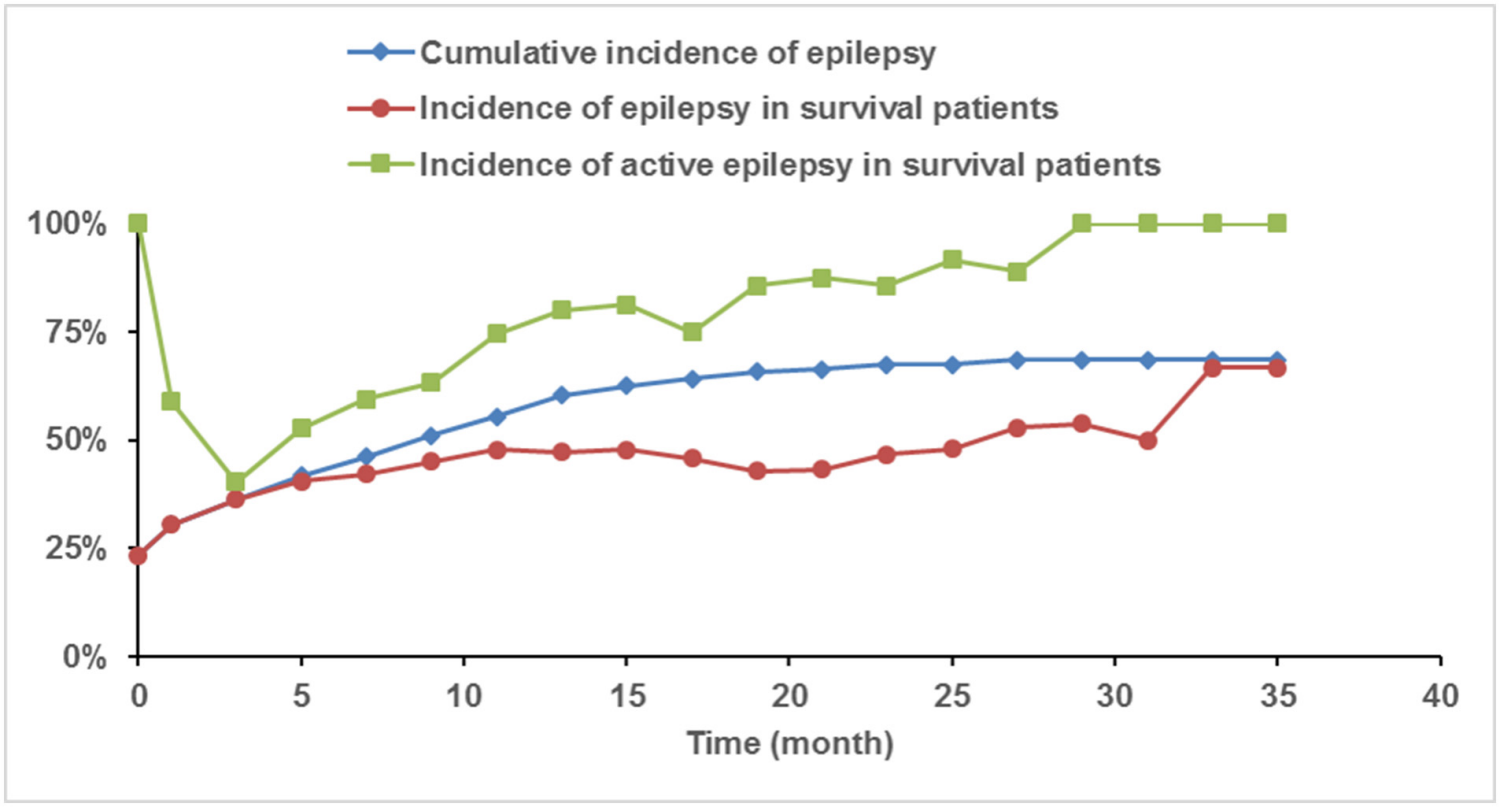
The Incidence of Epilepsy and Active Epilepsy in Patients with GBM. The cumulative incidence of epilepsy (solid black) before the initial resection (0 month) in patients with GBM, the incidence of epilepsy (red), and the incidence of active epilepsy (dashed black) in patients with epilepsy are shown at throughout disease progression following initial resection.

The incidence of epilepsy throughout disease progression is depicted in Figs 1 and 2. There were 43 patients (23.4%) with epilepsy before the initial resection. Eighty-three (45.1%) patients developed new-onset epilepsy, of which 38 cases developed 1 to 6 months following initial resection, 29 between 7–12 months post-resection, and 16 patients after 12 months post resection. The average time of seizure onset was 8.34±6.42 months post-resection, with the latest recorded point of onset at 27 months following initial resection. In total, 126 (68.5%) patients suffered epilepsy during the course of their illness.

Among the patients with pre-resection epilepsy, the percentage of active epilepsy rose from 40.3% at 3 months post-resection to 80.0% at 13 months post-resection. This trend continued such that 100% of patients with epilepsy had active disease by 29 months post-resection.

Of the first seizures experienced, 23 (18.3%) were simple partial seizures, 26 (20.6%) were complex partial seizures, and 77 (61.1%) were partial and secondary generalized seizures.

### Factors influencing the incidence of epilepsy

No significant differences in the incidence of epilepsy were found based on gender, age at initial resection, or size of the GBM. However, as expected, significant differences in the incidence of epilepsy were found based on the main location of the lesion. Of the 38 GBM patients with temporal lobe lesions, 18 (47.4%) suffered from epilepsy pre-operatively. In addition, of the 26 GBM patients with parietal lobe lesions, 9 (34.6%) experienced seizures pre-operatively. Thus, temporal or parietal lobe lesions are the greatest risk factors for preoperative epilepsy in GBM patients.

High rates of postoperative epilepsy were also observed in these patients, with 15 (39.5%) of the subjects with temporal lobe lesions and 9 (34.6%) of those with parietal lobe lesions experiencing post-operative onset epilepsy. However, the incidence of postoperative epilepsy was highest in the patients with frontal lobe lesions, with 38 of 61 patients (62.3%) developing seizures (Table 3).

**Table 3:** Resection and Recurrence by Lesion Location Between AED and non-AED Groups.

Tumor Location	Patients Who Received AEDs			Patients Who Did Not Receive AED		
	n	GTR	RT	n	GTR	RT
Midline/Bilateral	16 (21.9%)	2 (6.7%)	7.88±3.01	8 (11.8%)	4 (12.1%)	6.88±2.59
Thalamus	6 (8.2%)	0 (0.0%)	6.67±3.93	12 (17.6%)	1 (3.0%)	5.33±2.64
Frontal lobe	24 (32.9%)	12 (40.0%)	8.04±3.74	25 (36.8%)	12 (36.4%)	6.16±3.34
Temporal lobe	10 (13.7%)	8 (26.7%)	6.70±3.20	10 (14.7%)	9 (27.3%)	5.50±3.31
Parietal lobe	8 (11.0%)	3 (10.0%)	8.38±3.83	9 (13.2%)	3 (9.1%)	5.56±2.70
Occipital lobe	9 (12.3%)	5 (16.7%)	7.89±2.52	4 (5.9%)	4 (12.1%)	6.25±3.86
**Total**	**73**	**30**	**7.73±3.35**	**68**	**33**	**5.93±3.01**

GTR, gross total resection (P = 0.4730); RT, recurrence time (months; P = 0.0010).

Ten of 43 (23.3%) patients with preoperative epilepsy presented with one or more seizures in the first month following initial resection (Table 4). This compares to 13 of 141(9.2%) cases of active new onset epilepsy in the patient group without seizure history. The different adjunctive treatments used in the management of each patient had no discernable effect on the incidence of new-onset epilepsy following initial resection (Tables 5 and 6). As such, no significant difference in post-resection epilepsy rates was noted between patients who underwent 3D-conformal radiotherapy versus those treated by I^131^ intra-tumor radiotherapy or between patients receiving nimustine chemotherapy versus those receiving temozolomide. However, patients whose initial operations achieved gross total resections were much more likely to be seizure-free than those who received major or partial resections. Of the 43 patients with preoperative epilepsy, 16 of the 20 (80%) who underwent gross total resections were seizure-free at one month follow-up, compared with 7 of the 23 (30.4%) partial resection patients (Table 4, *P* = 0.0032).

**Table 4:** Effect of AEDs on Preoperative Epilepsy at 1 month Follow-up.

Therapy	Patients with Pre- and Postoperative Epilepsy	Postoperative Seizure-Free Patients	*P* Value
No. of Patients	20	23	
Gross Total Resection			0.0032
Yes	4 (20.0%)	16 (69.6%)	
No	16 (80.0%)	7 (30.4%)	
Radiotherapy			0.4629
I^131^	9 (60.0%)	14 (47.8%)	
3D-conformal	11 (45.0%)	9 (81.8%)	
Chemotherapy			0.2193
Nimustine	15 (75.0%)	12 (52.2%)	
Temozolomide	5 (25.0%)	11 (47.8%)	

**Table 5:** Effects of Antineoplastic Agents on Postoperative Onset Epilepsy in Preoperative Seizure-Free Patients.

Therapy	Patients with Postoperative Onset Epilepsy	Postoperative Seizure-Free Patients	*P* Value
No. of Patients	83	58	
Number of Resections			0.7994
One	10 (12.0%)	9 (15.5%)	
Two	54 (65.1%)	35 (60.3%)	
Three	19 (22.9%)	14 (24.2%)	
Extent of First Resection			0.6725
Gross total	48 (57.8%)	35 (60.3%)	
Major or Partial	35 (42.2%)	22 (39.7%)	
Radiotherapy			0.6262
I^131^	39 (47.0%)	24 (41.2%)	
3D-conformal	44 (53.0%)	34 (58.8%)	
Chemotherapy			0.1587
Nimustine	47 (51.8%)	25 (43.1%)	
Temozolomide	36 (48.2%)	33 (56.9%)	

**Table 6:** Seizure Occurrence Following AED Withdrawal in Patients in the New Onset Postoperative Epilepsy Prophylaxis Group.

AED Administration Duration	Patients who Developed Seizures Following AED Withdrawal	Patients who Remained Seizure-free Following AED Withdrawal
1 month	4	6
2–6 months	5	12
7–12 months	8	18

AEDs, anti-epilepsy drugs. Excludes patients who developed seizures during AED administration.

### Prophylactic AEDs for new-onset epilepsy

**Fig 3:**
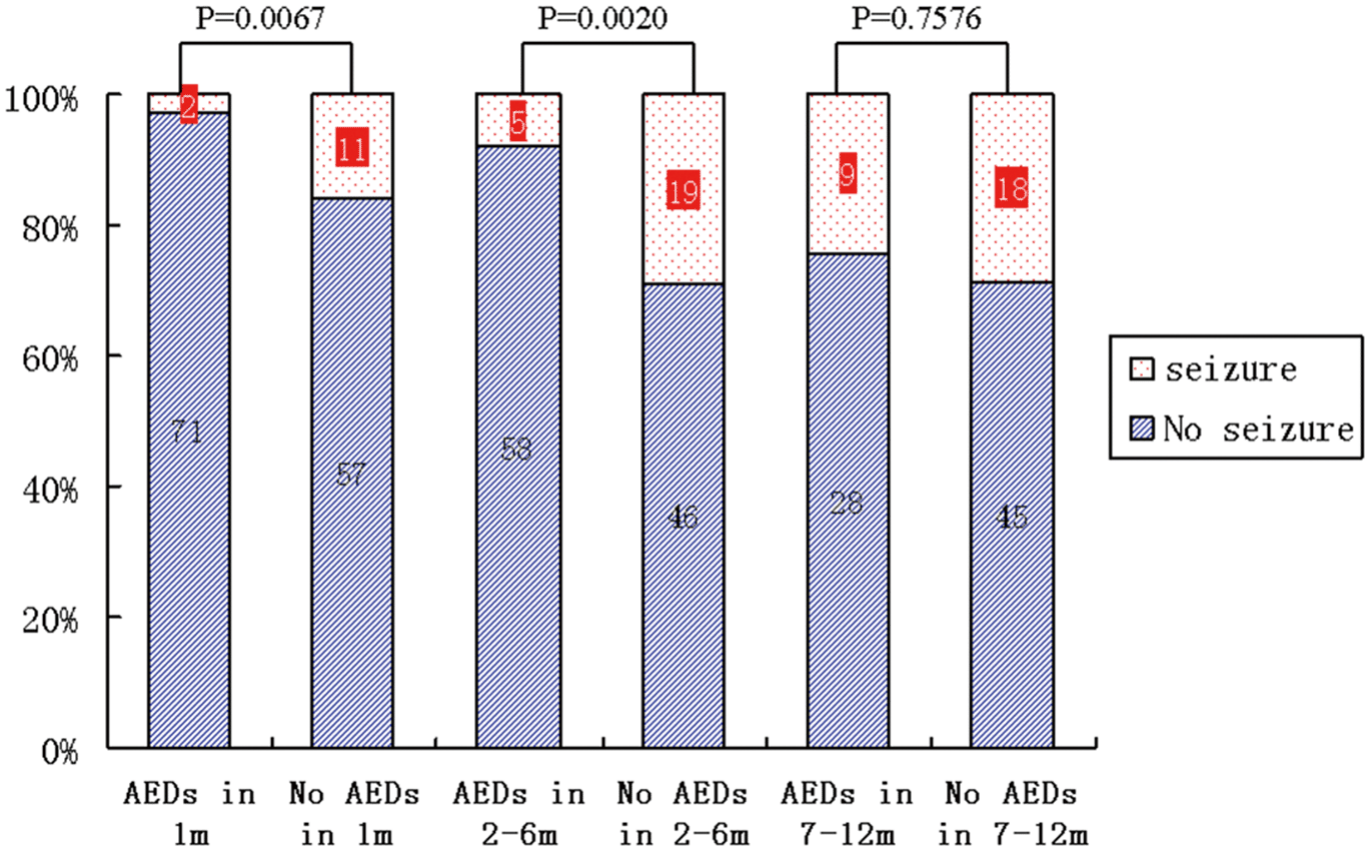
Antiepileptic Drug Administration for New Onset Postoperative Epilepsy Prophylaxis. Seizure occurrence over time in both AED prophylaxis and control groups.

Of the 141 patients without preoperative epilepsy, 73 received AEDs as a form of prophylaxis. As this is a retrospective study, these patients were administered prophylaxis on a case-by-case basis based on the practice of individual physicians and were not representative of overall patient demographics. The patients with prophylactic AEDs had significantly larger tumor sizes and reached significantly longer postoperative survival times than those without prophylactic AEDS (Table 7). Patients who were prescribed AEDs had a lower incidence of post-operative epilepsy before the 6 month follow-up (Fig 3). There was a significant difference in the incidence of epilepsy at the 1 month follow-up between those prescribed and those not prescribed AEDs (*P* = 0.0067), as well as at follow-ups at 2 through 6 months (Fig 3). No statistically significant difference was observed during follow-up at 7 through 12 months (Table 6).

**Table 7:** AEDs for New Onset Postoperative Epilepsy Prophylaxis.

Postoperative Duration	Patients with Postoperative Onset Epilepsy	Seizure-Free Patients	*P* Value
1 month	13	128	0.0067
AEDs	2 (15.4%)	71 (55.5%)	
No AED	11 (84.6%)	57 (44.5%)	
2–6 months^a^	24	102	0.0055
AEDs	5 (22.7%)	56 (54.9%)	
No AED	19 (77.3%)	46 (45.1%)	
7–12 months^b^	22	65	0.6283
AEDs	9 (40.9%)	26(40.0%)	
No AED	13 (59.1%)	39 (60.0%)	

AEDs, anti-epilepsy drugs.

^a^ Excluding patients with survival times of less than 6 months and without epilepsy at 1 month follow-up.

^b^ Excluding patients with survival time less than 12 months and without epilepsy at 6 month follow-up.

### Effect of epilepsy on karnofsky performance score

Forty-three patients survived for 18~24 months after initial resection and 20 of these patients developed epilepsy before 12 months. No significant difference in survival time and age at first resection was found between the 20 patients with epilepsy and the 23 without. The mean Karnofsky score was 47.25±10.94 in patients with epilepsy at 12 months follow-up, which was significantly lower than 55.65±13.76—the mean score for those without epilepsy (*P* = 0.0341).

## Discussion

In this study, the incidence of epilepsy before initial resection was 23.4% in patients with GBM, which is within the range that has been demonstrated in literature with primary brain tumors. [1] Furthermore, the cumulative incidence of epilepsy in patients with GBM was 68.48%—considerably higher than has been previously reported—and responded poorly overall to AEDs. [1] Although multiple AEDs were used, disease activity rose from 40.3% of patients with epilepsy at 3 months to 80.0% at 13 months. One hundred percent of patients suffered from active epilepsy by 29–35 months follow-up. Additionally, the difference in Karnofsky scores between the patients with and without epilepsy was slight but significant. This result demonstrates the decreased performance of patients with epilepsy and corroborates the decreased quality of life observed in epileptic patients, which possibly results from the structural and functional consequences of frequent seizures and patient anxiety with regard to future seizures. Additionally, the ineffective use of AEDs and their associated side effects in patients at or after 7 months post-resection may further reduce quality of life. [1, 11] The data presented herein highlights the relatively common nature of epilepsy with GBM and the need for its proper evaluation and treatment. [9]

Induction of epilepsy in GBM patients can be attributed to an imbalance of inhibitory and excitatory neural networks, which is caused by invasion and destruction of normal tissue and neural connections. [1] Complex structural, molecular, or metabolic changes within the tumor can affect incidence rates, and rapid growth of GBM can result in hypoxia of the surrounding nervous tissue, promoting focal epileptogenesis and exacerbating already present seizures. [1, 7] Additionally, low levels of glutamine synthetase are correlated with both incidence of epilepsy and increased survival in GBM patients. [12–14] Of the patients in this study, gross total resection of GBM rendered 80% of patients with preoperative epilepsy seizure free in the first month following the initial operation. This result supports prior research that associated total resection with better seizure outcomes in patients with lower-grade gliomas. [1, 15]

We found the incidence of epilepsy to be higher among patients with tumors located in the temporal (86.8%) and frontal (82.0%) lobes, corroborating the 2009 findings by Chaichana et al. [9] This was higher than the incidence for lesions in all other areas and is consistent with previous findings that show the epileptogenic zone in secondary neocortex epilepsy is usually located within the frontal and/or temporal areas. [16]

GBM is characterized by frequent tumor recurrence, and the development of post-resection epilepsy has been previously linked to tumor progression. [9] It is important to note that the latest onset of epilepsy occurred 27 months following initial resection and 3 patients demonstrated no seizure activity whatsoever until death at 35 months. This may be due to an epileptic susceptibility or gene that may have played a role in epileptogenesis, as has been previously suggested. [1, 6–7]

The consensus within the literature suggests that AEDs should be utilized at the first presentation of epilepsy in patients with GBM. [13, 17–19] However, the prophylactic use of AEDs in seizure-free GBM patients remains controversial. A meta-analysis of prophylactic AED use in patients with brain tumors between 1966 and 2007 and a report by the Quality Standards subcommittee of the American Academy of Neurology both indicated that there was no benefit from prophylaxis. [20–21] These studies did not recommend the routine use of AEDs for seizure prophylaxis and suggest they be withdrawn within the first week after surgery if patients do not develop seizures. [18] Despite this, a recent paper surveying neurosurgical practitioners showed that 70% of neurosurgeons choose to administer prophylactic AEDs in patients with brain tumors. [19]

Though the data reported herein are limited by the retrospective nature of the study, our results—in contrast to the present literature—suggest that prophylactic AEDs can reduce postoperative epilepsy in the first month after initial resection. This observation remained at postoperative follow-ups at 2 through 6 months. Beyond 7 months, the prophylactic benefit of AEDs was not evident. Furthermore, the percentage of patients with GBM undergoing total resection was low and the rate of recurrence and reoperation was high in the first 6 months following initial resection. Finally, this study lacks the large number of patients that would need to be randomized in order to more clearly show the benefit of prophylaxis.

No drugs are without side effects, and consideration should be given to those of AEDs. [8] However, due to recent pharmaceutical advances, side effect profiles have been greatly reduced. Levetiracetam, one such example of the new generation of AEDs, which has scarce side effects and does not interact with common chemotherapeutic agents. [5, 22–25] A recently published literature review indicates that in the last decade there have been no randomized prospective studies evaluating the utility of second- and third-generation AEDs in preventing epileptogenesis in glioma patients. [1] We therefore recommend a randomized prospective study on the use of a prophylactic AEDs, especially levetiracetam, for the first 6 months following the resection of GBM in order to further verify our findings.
